# Characterising differential vulnerability of neurons to tau‐mediated toxicity

**DOI:** 10.1002/alz70855_100390

**Published:** 2025-12-23

**Authors:** Lovesha Sivanantharajah, Yelena Ivanova, Amritpal Mudher, David Shepherd

**Affiliations:** ^1^ Bangor University, Bangor, Gwynedd, United Kingdom; ^2^ University of Southampton, Southampton, United Kingdom

## Abstract

**Background:**

A key feature of tauopathies, including Alzheimer's Disease (AD), is progressive deterioration of neuronal subsets within specific brain regions. One explanation for regional preference is selective vulnerability of different neurons to pathogenic proteins. To date, animal models have examined pathogenic protein expression in broad or heterogeneous neuronal populations, meaning noted pathological hallmarks represent an average of disease phenotypes over many neuron types. Our solution is a genetic toolset for targeting gene expression to single neuron types in the *Drosophila* central nervous system (CNS), allowing us to analyse disease pathogenesis and progression over lifespan at sub‐cellular resolution.

**Method:**

Using this toolset, we analysed the vulnerability of adult neuron types in the *Drosophila* CNS to the toxicity of the highly‐phosphorylated human tau isoform, hTau0N3R. We examined the effects of tau on cell morphology, pathological tau phosphorylation (AT8), and trafficking (vesicles and mitochondria).

**Result:**

Our model recapitulated typical age‐dependent tau phenotypes shown in many studies (e.g., loss of synaptic terminals, tau mislocalisation, beading/blebbing of neuronal processes). While all neurons examined were affected by tau, some were more vulnerable than others. The tau‐mediated pathogenic effects fell on a spectrum demonstrating that fly neurons are *differentially vulnerable* to tau pathology. Mechanistically, total tau levels did not correlate with vulnerability; rather, degeneration correlated with significant age‐dependent increases in phospho‐tau levels in the same neuron type, and tau mislocalisation into dendrites. Lastly, increased *p*‐tau levels correlated with downstream vesicular and mitochondrial trafficking defects in some neurons but not all.

**Conclusion:**

This work highlights the heterogeneity of responses underpinning neuronal vulnerability to tau. Thus, innate cellular factors play an essential role in determined vulnerability. Further, this work provides a new model for studying AD with sub‐cellular resolution. Current ongoing work aims to examine the role of individual tau isoforms on selective vulnerability.

